# Digital Sorting of Pure Cell Populations Enables Unambiguous Genetic Analysis of Heterogeneous Formalin-Fixed Paraffin-Embedded Tumors by Next Generation Sequencing

**DOI:** 10.1038/srep20944

**Published:** 2016-02-11

**Authors:** Chiara Bolognesi, Claudio Forcato, Genny Buson, Francesca Fontana, Chiara Mangano, Anna Doffini, Valeria Sero, Rossana Lanzellotto, Giulio Signorini, Alex Calanca, Maximilian Sergio, Rita Romano, Stefano Gianni, Gianni Medoro, Giuseppe Giorgini, Hans Morreau, Massimo Barberis, Willem E. Corver, Nicolò Manaresi

**Affiliations:** 1 Silicon Biosystems S.p.A, Bologna, Italy; 2 Department of Pathology, Leiden University Medical Center, Leiden, Netherlands; 3 European Institute of Oncology, Milan, Italy

## Abstract

Precision medicine in oncology requires an accurate characterization of a tumor molecular profile for patient stratification. Though targeted deep sequencing is an effective tool to detect the presence of somatic sequence variants, a significant number of patient specimens do not meet the requirements needed for routine clinical application. Analysis is hindered by contamination of normal cells and inherent tumor heterogeneity, compounded with challenges of dealing with minute amounts of tissue and DNA damages common in formalin-fixed paraffin-embedded (FFPE) specimens. Here we present an innovative workflow using DEPArray™ system, a microchip-based digital sorter to achieve 100%-pure, homogenous subpopulations of cells from FFPE samples. Cells are distinguished by fluorescently labeled antibodies and DNA content. The ability to address tumor heterogeneity enables unambiguous determination of true-positive sequence variants, loss-of-heterozygosity as well as copy number variants. The proposed strategy overcomes the inherent trade-offs made between sensitivity and specificity in detecting genetic variants from a mixed population, thus rescuing for analysis even the smaller clinical samples with low tumor cellularity.

Formalin-fixed, paraffin-embedded (FFPE) tissue samples are the *de-facto* standard in pathology labs. Using FFPE tissue for tumor genomic profiling by next generation sequencing (NGS), as required by the implementation of precision medicine, can be challenging due to the contaminating stromal cells and low percentages of tumor cells in the sample. Further, background of normal diploid cells dilutes the signal associated with quantitative genomic features like copy number variations (CNVs), loss of heterozygosity (LOH) and simple homozygous/heterozygous status of a variant in a tumor cell subpopulation. This strongly impairs their accurate detection by NGS, and limits CNV analysis to samples having relatively high tumor-content and high tumor-gene amplification levels. In addition, prevalence of a mutation within the tumor cannot be assessed accurately, which may skew its evaluation as a putative biomarker. Thus, depending on the molecular assay, different cut-offs for tumor content are used, e.g. 20–30%^1^, 45%^2^, 70%^3^, but, invariably, insufficient tumor fraction remains a major cause precluding molecular analysis (e.g. up to 15%^1^).

Sequencing artifacts, induced by DNA damages associated with FFPE preparation protocol^4^,^5^ together with tumor heterogeneity^6^ further exacerbate the technical difficulties of identifying the presence of true somatic alterations in a sensitive and specific way. These challenges are relevant across a broad range of specialties, including biomarkers discovery, patients enrollment for clinical trials, and in routine clinical use to inform therapy management.

Microdissection is often used to enrich for tumor cell fraction, but the approach is inherently labor intensive and purity is limited, especially when tumor cells are highly intermingled with stromal cells, e.g. inflammatory cells or cancer associated fibroblasts. FFPE tumor tissues can be disaggregated to be transformed into individual cell suspensions and stained for cytoskeleton proteins and DNA content, allowing fluorescence-based cytometric analysis^7^. However, molecular pathology labs are often faced with small size samples, as a result of the biopsy procedure and/or the scant availability of tissue leftover from the necessary additional histopathological analyses or as a result of a previous neo-adjuvant therapy. This limitation prevents the use of conventional FACS sorters that require relatively high cell numbers.

The recent introduction of DEPArray™ technology (Silicon Biosystems)^8^ for image-based dielectrophoretic (DEP) cell sorting has enabled the automatic isolation of pure cells from low-count cell suspensions from very small samples. Successful isolation and genetic characterization of individual pure circulating tumor cells (CTCs) from enriched peripheral blood^9^,^10^ have been demonstrated.

Here we present for the first time an innovative workflow, including FFPE tissue disaggregation and fluorescent staining combined with DEPArray™ sorting, enabling isolation of pure tumor cells for unambiguous genetic analysis results demonstrated with targeted NGS assays. We apply it on minute, 0.6 mm diameter FFPE tissue cores as a model for clinical biopsies and tissue rolls as a model for archival samples.

## Results

### Detection, isolation and recovery of pure tumor cell subpopulations by DEPArray™ sorting technology

**Figure 1:**
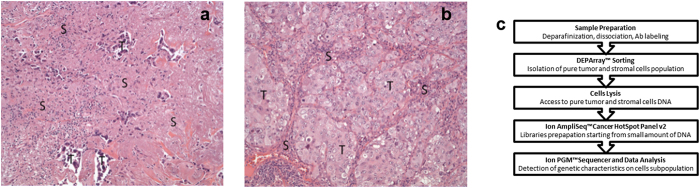
(**a,b**) H&E staining of paraffin sections from sample S10 (5% tumor cellularity) and S09 (40% tumor cellularity), respectively. Fifty μm sections or 0,6 mm diameter core punches were collected from FFPE tissue blocks. Paraffin sections taken from all samples were H&E stained to facilitate identification of the tumor area of interest. The slide was used to guide enrichment of carcinoma and carcinoma-associated stroma by trimming the normal epithelium and/or superfluous stromal tissue. Images were acquired using Leica DMA 600 B microscope, equipped with 10× objective and DFC280 camera. Abbreviations: S = stroma, T = tumor. (**c**) Schematic overview of the workflow used in the study.

A total of 23 formalin-fixed paraffin-embedded samples from 23 patients were obtained from the tissue bank of the Pathology Department, Leiden University Medical Center and from European Institute of Oncology, Milan, according to the medical ethical guidelines described in the Code for Proper Secondary Use of Human Tissue established by the Dutch Federation of Medical Science (www.federa.org) and IEO ethical committee internal procedures, respectively. The series included 50 μm FFPE tissue sections from ovarian (n = 1), pancreatic (n = 2), and 0.6 mm diameter FFPE tissue cores from lung (n = 9) and colon adenocarcinoma (n = 11), collected between 1993 and 2014. Estimated tumor cellularity ranged between 5% and 60% (Table 1 and Fig. 1).

Small FFPE tissue cores taken from representative tumor areas are used routinely in pathology. This technique enriches for tumor cells in highly intermingled samples and is much less laborious than laser guided microdissection. Data from small FFPE tissue cores of highly intermingled clinical sample are presented. Cell suspensions were prepared with a modified protocol based on the procedure described by Corver and Haar^11^.

A small amount of the labeled cell suspension was washed twice with 1 ml of SB115 buffer (Silicon Biosystems) by centrifugation at 1000 g for 5 min. Pellet was resuspended in the same buffer in order to have 400–800 cells/μl. DEPArray™ A300K cartridge (Silicon Biosystems) was loaded with 830 μl of SB115 buffer and 13 μl of sample.

Cells sorting was executed according to DEPArray™ User’s Manual rev 1.1_sw 2.0.1. We implemented a specific workflow for FFPE application: Cage Parameters, Chip Scan Setting and Sorting Mode are automatically uploaded by clicking few buttons. More specifically, the Cage Parameters include selection of the electrode pattern creating more than 30,000 DEP cages in the main chamber of DEPArray™ chip. Cells are trapped in stable levitation within DEP cages during image acquisition and analysis by the embedded optical fluorescence microscope.

Chip Scan is carried out acquiring the following channels: bright-field, DAPI, FITC (for Alexa488), APC (for Alexa647). Events are detected automatically in DAPI. From the images acquired in each channel a region-of-interest (ROI) is computed for the event. The following parameters are measured and stored in the *particle database*: mean, max and integral intensity computed over the ROI, as well as morphological parameters from the ROI itself, including area, roundness and diameter.

Qualitative and quantitative marker evaluation, along with cell DNA content measurement, was performed with the CellBrowser™ analysis software integrated in DEPArray™ system by which it is possible to view and select cells from the *particle database* according to multiple criteria and then assign the particles of interest to user-defined groups.

All the analysis tools (scatter plots, histograms, selection groups, image panels), can be combined so that the output subpopulation of a tool can be used as input population for another tool.

**Figure 2:**
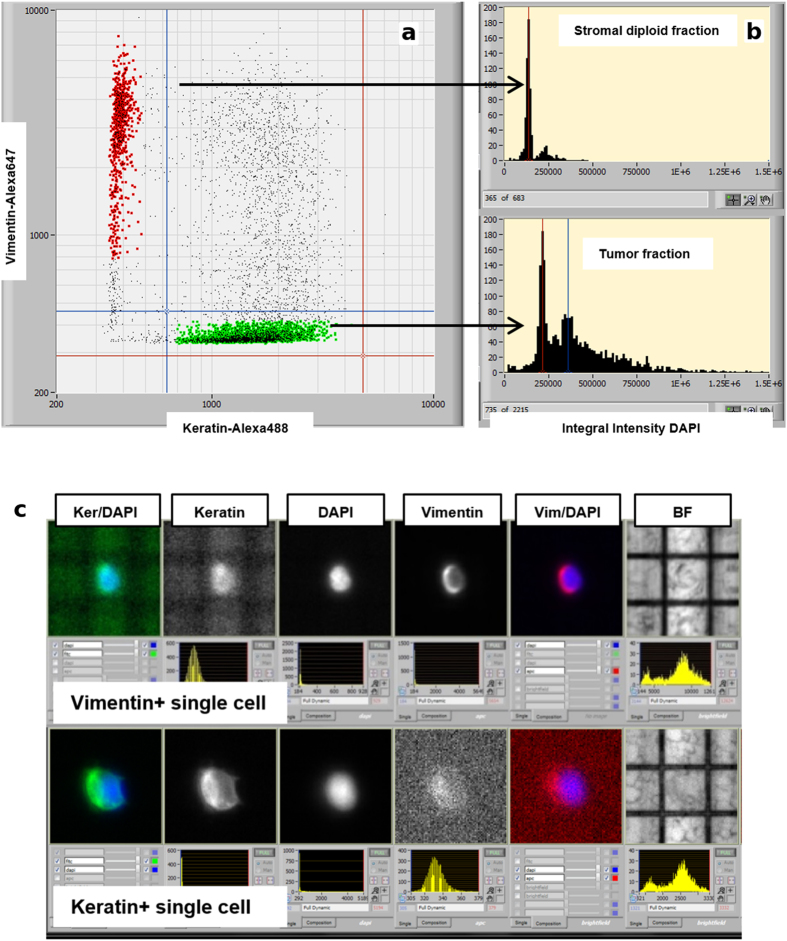
DEPArray™ analysis and gating based on scatter plots, histogram and images. (**a**) Simultaneous detection of two distinct cell populations based on antigen expression from a dissociated FFPE specimen by using the mean intensity keratin-Alexa488 vs vimentin-Alexa647 scatter plot. (**b**) After gating for the vimentin-positive (V + ) and keratin-positive (K + ) cell populations, single-parameter DNA histograms are generated. The V + fraction is used as an internal DNA-diploid reference for K + population DNA content assessment. (**c**) Images of individual events can be viewed on an image bar, allowing cells of interest to be identified and flagged for recovery.

As a first step, all population is filtered to select particles captured in the dielectrophoretic field-cages, i.e. suitable for the cell routing, and the frequency distribution of particles based on the selected parameter is showed in a “in cage” histogram. The selection of “in cage” events is then used as input in a scatterplot of mean intensity keratin-Alexa488 versus mean intensity vimentin-Alexa647 (Fig. 2a).

Despite the very low cell count for each of the input samples, the detected number of events was enough to identify the main subpopulations. Notably, we were able to compensate sample-to-sample variations in the intensity of fluorescence markers by adjusting *a-posteriori* the gating of subpopulations, i.e. after scanning the available sample.

Once “V + K-” (Alexa488-/Alexa647 + /DAPI + ) and “K + V-” (Alexa488 + /Alexa647-/DAPI + ) populations are identified, a gating is applied on particles that meet the desired characteristics and the two groups are saved for the following comparative analysis of their DNA content in an integral-intensity DAPI histogram analyzing the number of peaks and their position.

The V + K- fraction is used as an internal DNA-diploid reference for keratin positive fraction DNA content assessment. Abnormal DNA content has an impact on peak position and multiple populations with different DNA contents can be identified: DNA diploid, DNA near-diploid and DNA hyper-diploid fraction.

Subpopulations of interest are selected by gating the cells according to their DNA content peak (Fig. 2b) and assigned to the appropriate group. Finally, we refined the cell selection by reviewing the image-gallery provided by the CellBrowser™ (Fig. 2c). By visual inspection we discarded doublets, clumps and spurious events while ensuring recovery of only intact, individual cells exhibiting the desired fluorescence patterns and DNA content.

Steps needed for Cell Routing and Recovery are activated by Recovery Manager™ software. Cells selected with the CellBrowser™ are automatically moved across the chip active area, from the Main Chamber to the Park Chamber first and then to Exit Chamber to be recovered.

The Park Chamber, for the FFPE sorting mode, has a total of 680 available parking positions whereas the Exit Chamber has 507 parking positions, therefore the maximum number of cells in a single recovery is 507 cells (if necessary, more recoveries can be collected in the same tube with a special procedure). Depending on the number of selection groups, the total number of cells per group is subject to certain limitations.

By DEPArray™ sorting, precise numbers (mean = 133, median 98, range = 5–600) of pure homogenous cells from the major populations of tumor and contaminant diploid stromal cells, as well as other minority putative tumor cells positive for both keratin and vimentin (K + V + ), were recovered.

In order to reduce the collected volume of SB115 buffer to 1 μl of PBS required for downstream analysis, each pool (n = 115) of recovered cells was centrifuged at 14,100 g for 10 min in a fixed rotor centrifuge. Seventy μl of buffer were removed, 100 μl of PBS were added without disturbing the sample pellet and tubes were centrifuged at 14,100 g for 10 min in a fixed rotor centrifuge. To reach the final volume of 1 μl containing the isolated cells, a 200 μl pipette with its tip was pointed to the tube wall opposite to the centrifugation direction. All buffer volume was aspirated carefully while sliding the tip on the tube wall and following the air-liquid meniscus toward the tube bottom without dipping the tip. Cells were lysed using SB LysePrep™ Kit (Silicon Biosystems) adding the reagents into the same tube.

Since the number of cells recovered is digitally controlled by DEPArray™ system, there is no need for DNA quantitation. IonTorrent AmpliSeq™ Cancer Hotspot Panel v2 reagents were directly added to the same tube and thermal cycling program was adjusted according to the number of cells. In addition, for all processed samples we generated libraries from unsorted disaggregated cells (n = 28, pool of 300–1000). All successful libraries (n = 114) were sequenced with the IonTorrent™ PGM with mean coverage ranging from 800× to 3100× (median 2100×) and sequencing data were processed with Torrent Suite (Supplementary Table S1), with “*somatic low-stringency”* variant calling parameter configuration.

### Pure cell separation produces unambiguous NGS results

The synoptic comparison of the variant frequencies between pure, homogenous cell subpopulations allowed us to assign unambiguously genetic variants to different classes, clearly separating from background noise and artifacts.

**Figure 3:**
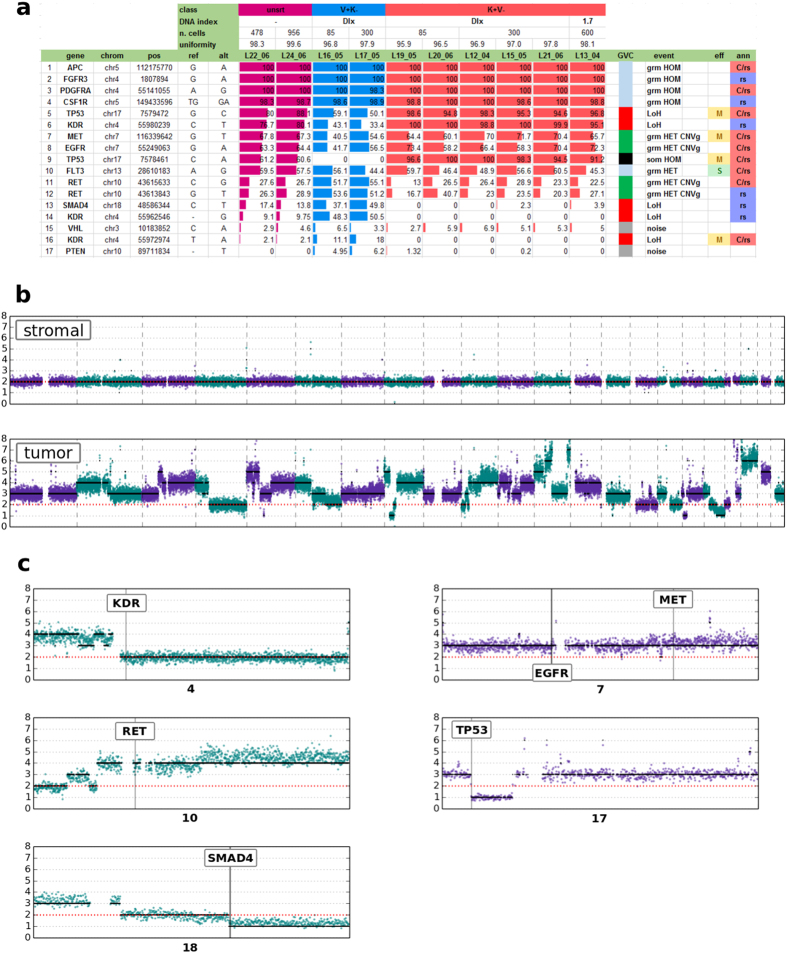
Summary of variant frequencies and copy-number profiles obtained by low-pass whole genome sequencing in S01 patient, affected by an ovarian cancer. (**a**) Only a subset of significant variants are displayed. Table shows variants in rows and cell populations in columns (unsrt, unsorted cells; V + K-DIx, stromal cells with mixed ploidy; K + V-DI = 1.7, tumor cells with a DNA index of 1.7 related to stromal peak; K + V-DIx, tumor cells with mixed DNA content). Variants for each cell population are represented by a cell box filled with different colors based on relative frequency: red for tumor, blue for stromal, magenta for unsorted. In the table header, information about number of cells and sequencing uniformity are present. The frequency patterns highlight different kind of variant events summarized in a colored box (column GVC, Genetic Variant Class): somatic homozygous variant (black), copy number gains (green), LOH (red), germline variants (ice), and background-noise (gray). In the right-side, the “eff” column describes gene/protein effect where missense mutations (M) are marked with a yellow square and splice variants (S) with a green square; empty cells means no protein effect (intron or synonymous variant). Variants annotated in COSMIC (C) and/or dbSNP (rs) databases are represented in “ann” column with respectively a red and blue box. (**b**) Whole-genome profiles (chr1-22) of stromal (top) and tumor (bottom) populations. Ploidy values are indicated in the y-axis, with the diploid state highlighted with a red dotted line; in the x-axis, the alternation of different chromosomes are plotted with different colors. Thanks to information given by DNA index, tumor profile is centered on ploidy = 3, as opposite to diploid stromal profile. (**c**) CNV profiles of specific chromosomes were enlarged to better display the copy-number of specific genes that experience, according to targeted panel data (Fig. 3a), LOH and CNV events. At the bottom of the x-axis, the chromosome number is indicated.

Frequently, for loci harboring germline heterozygous Single Nucleotide Polymorphisms (SNPs) with variant frequencies around 50% for pure stromal cells, we readily detected loss of heterozygosis (LOH) in tumor cell populations as binary (0%/100%) variants. As shown in Fig. 3a, the variant calls of *SMAD4* (row 13) and *TP53* (row 5) show the frequency pattern generated by a deletion of one allele in cancer cells: in the first case, the loss of the variant allele will decrease to zero the frequency; in the second case, the 100% frequency indicates the loss of the wild-type allele.

Moreover, we detected somatic mutations with 100% variant frequency, only observable as heterozygous in the unsorted samples and as wild-type in stromal cells from the same patient, confirming 100% purity of sorted cells. The real zygosity state of the known missense *TP53* mutation (Fig. 3a row 9) is only detected by sorted tumor cells, emphasizing an important genetic feature that would otherwise be hidden. In fact, the absence of wild-type sequence, can lead to complete loss of function in the cells as only the mutant protein is present.

Dealing with populations of pure cells, quantitative genomic features such as copy number gains and losses were also reproducibly identified in tumor cell replicates as deviations from the 0%/50%/100% frequencies typical of germline variants detected in stromal cells. The *MET* and *RET* mutations (Fig. 3a row 7 and 12, respectively) describe two distinct gain events on tumor replicates, revealing a gain of the variant allele in the first and a gain of the wild-type allele in the second case.

Germline heterozygous and homozygous variants are characterized by 50% and 100% frequencies, respectively, for both tumor and stromal cells. The *FLT3* variant (Fig. 3a row 10) shows a heterozygous state. Also, several homozygous germline variants can be observed in *APC, CSF1R, FGFR3* and *PDGFRA* genes (Fig. 3a row 1–4).

A notable exception to what we described above is the *KDR* variant (Fig. 3a row 16) corresponding to a known variant annotated in dbSNP and COSMIC (rs1870377, COSM149673) which deviates from the expected 50% variant frequency. This result can be interpreted as deriving from a PCR amplification artefact for that specific amplicon. We speculate that this may be linked to the presence of some SNPs in correspondence of the target amplification primers (Supplementary Fig. S1), which in turn can cause the preferential amplification of the wild-type allele thus reducing the variant allele representation. The absence of the variant allele in tumor cell recoveries indeed is coherent with the observation of the LOH event in the same *KDR* gene, revealed by two additional germline heterozygous SNPs in the stromal cells, flipping in tumor cells to either 0% or 100% variant in tumor cells (Fig. 3a row 14 and row 6, respectively).

Furthermore, several variants were called at low frequency regardless of the cell population considered. These variants can be attributed to background noise due to artefacts related to the damages induced by FFPE sample preparation^4^,^5^ or sequencing errors connected to the target enrichment^12^ or the sequencing technology. In particular, these variants would often be substantially undistinguishable from true somatic variants diluted by the normal wild-type cells if one considers only the unsorted fraction. Cell purity also boosts variant frequency of true positives, enabling easy distinction from false positives. For example, in a pancreatic sample (Supplementary Fig. S2) a *KRAS* and a *TP53* somatic mutations were barely detectable (3.1%, 2.8% respectively) in the unsorted fraction, while the K + V- fraction had about 6–10× higher variant frequency (18.2%, 30.1%).

In summary, the data show clearly that deep-sequencing of unsorted fractions gives a blurred picture, often misleading, of the genetic profiles of the different subpopulations. By contrast, DEPArray™ sorting provides a clean separation of their different molecular features (Supplementary Table S3).

### Copy number assessment by low-pass whole-genome sequencing

Whole-genome sequencing (WGS) is an established method for the discovery and detection of alterations in chromosomal copy number. Useful copy-number profiles can be obtained also with a low coverage (about 0.1×)^13^,^14^. In order to confirm the predicted LOH and copy-number events detected with targeted panel and depicted in Fig. 3a, a low-pass WGS was performed on both aneuploid (300 cells, DI = 1.53) tumor population of S01 sample and related stromal population, 126 cells (0.27× and 0.16× sequencing depth, respectively). As apparent in Fig. 3b, the diploid stromal population shows a flat profile consistent with normal diploid cells, whereas the triploid tumor population shows several somatic copy-number alterations in forms of gains and losses. This first observation allows to further demonstrate how the DEPArray™ sorting was able to resolve the heterogeneity and to obtain pure populations.

Moreover, low-pass WGS data can be used for confirmation of the CNV and LOH events predicted from targeted panel data.

For what concerns CNVs, in the panel table *MET* and *EGFR* genes have two variants (Fig. 3a-row 7,8) both with a mean frequency of 66%, consistent with a 1-copy gain of the mutant allele. Observing the low-pass profiles (Fig. 3c), *MET* and *EGFR* are both placed in chromosome 7 in a region with 3-copy alleles, as expected.

Furthermore, *RET* gene seems to have experienced 2-copy gains of the wild-type allele (Fig. 3a-row 11,12), as indicated by the mean 25% variant frequencies. This hypothesis is confirmed by the localization of *RET* gene in a 4-copy region of the genome (Fig. 3c).

As shown in Fig. 3c, *SMAD4* and *TP53* are placed at the beginning of a somatic loss alteration corresponding to 1-copy allele, easily explaining the LOH events (Fig. 3a-row 13 and row 5), leading to the binary (0/100%) variants, as well as the somatic homozygous mutation of *TP53* (Fig. 3a-row 9). Differently, *KDR* is located in a genome region with 2-copy alleles, suggesting a pair of mutational events, which led first to the loss of a copy and then to a subsequent gain of the residual copy, explaining the three independent LOH events (Fig. 3a-row 6,14,16) detected by the panel.

To assess further the advantage of DEPArray™ sorting on copy-number analysis, a low-pass WGS was performed also on tumor and stromal populations and on the unsorted fraction of S09 sample, characterized by a relatively low tumor cellularity (40%). As shown in Supplementary Fig. S3, in the low-pass profile of sorted pure tumor cells, numerous gains and losses are clearly identified along the genome. By contrast, in the unsorted sample the signal is diluted by the contamination of normal diploid cells, therefore all losses and most of the gains are missed.

Moreover, within DEPArray™ workflow we obtain a ploidy estimation of the recovered tumor cells, which can be input to the low-pass CNV algorithm to set appropriately the baseline. As shown previously^15^, this is important because the determination of DNA index influences the interpretation of the copy-number profiles.

### Evaluation of method applicability in relation to sample age and cell number limits

Although formalin-fixation is the most commonly used method in pathology departments for the storage of tumor tissue specimens, this process creates many problems in the context of genetic analysis, as it can cause fragmentation and/or induce mutations in the DNA material^16^. These damages are more visible when the amount of sequenced DNA template is lower, as in case of a low cell number.

Several factors can modulate the DNA damage mediated by formalin, such as fixation temperature, fixative buffer, time to fixation, storage duration and improper conditions of the pathology archives, in term of humidity and temperature. Most of these parameters are not tracked, making it difficult to assess their impact. Conversely, information on storage age is normally available, thus we analyzed the relationship between the rate of success/failure of sequencing libraries and the year the tissue was placed in storage.

**Figure 4:**
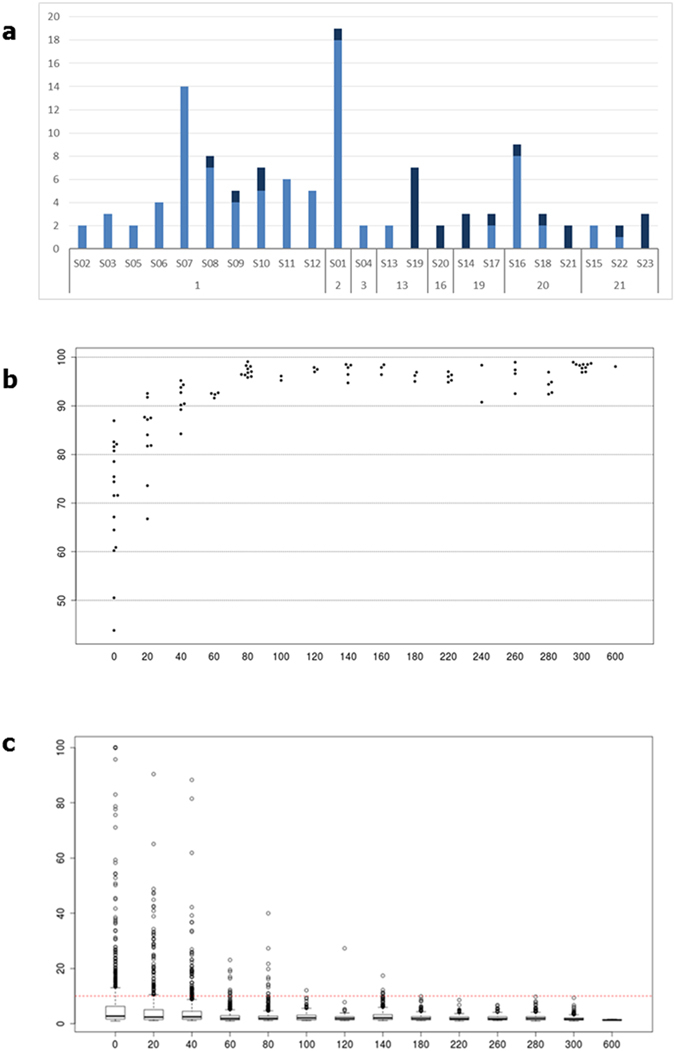
Evaluation of method applicability in relation to sample age and cell number limits. (**a**) Histogram showing the percentage of failed libraries in relation with samples and storage years (dark blue = failed library). (**b**) Analysis of coverage uniformity versus number of cells recovered. (**c**) Distribution of allele frequencies of “singleton” variants per bins of 20 cells. Red dashed line shows the 10% frequency threshold.

For 12 samples that had been stored for up to 3 years we obtained sequenceable libraries in 72 out of 77 (94%) sorted cell recoveries (cell number median = 85, range 5–600). For 11 older samples (storage age ranging 14–21 years), the success rate in library creation (cell number median = 158, range 15–297) dropped substantially (19/38 = 50%). As shown in Fig. 4a, it was apparent that the failed libraries did not distribute randomly but rather concentrated in specific older samples.

By contrast, we did not see a clear relationship between the number of cells recovered and the library-creation failure rate. Nevertheless, it was apparent that there is a relation between the number of cells recovered and coverage uniformity (Fig. 4b). We observed excellent coverage uniformity (mean = 97%) for recoveries (n = 53) in the range of 81–600 cells, even higher than the uniformity (91%) we observed by sequencing 10 ng of DNA (n = 2) obtained by a conventional FFPE extraction kit (QIAamp DNA FFPE Tissue Kit, Qiagen, Germany). Mean uniformity gradually decreased to 88% for cell recoveries (n = 21) in the range 21–80, and further decreased to 72% for lower cell numbers (n = 17). This trend is coherent with the compounded effect of DNA fragmentation due to FFPE process, which reduces the amount of template amenable for PCR, with the decrease in template copies due to the reduction of cells recovered^5^.

In turn, the reduction of template copies amplifiable by the target enrichment PCR increases the chance for formalin-induced DNA mutations to be over-represented in the amplified material, potentially causing false positive variant calls.

To study this aspect in more detail, we tried to assess putative false positives focusing on “singletons” within the whole set of variant calls across all sequenced libraries. We classified a variant as “singleton” if it is present only once in the subset of those samples with at least one replicate for the same cell population. In this way, a true positive variant should occur in at least two replicates and would thus be excluded from the “singleton” class. Moreover we did not considered “singleton” also those variants present in only one of two replicates but found at least once in another sequenced library. We considered those variants as potentially true because sometimes there are not enough reads in some regions of certain libraries. This is due to the poor quality of FFPE samples that sometimes can lead to a loss of some amplicons and to a resulting very-low/absent coverage, not sufficient for variant calling. Conversely, a variant called only once across all libraries is likely a false positive due to FFPE-related DNA damage.

These “singleton” variants were analyzed by grouping their frequency distribution by cell number (Fig. 4c). We observed that the variant frequency distribution is wider with a low number of cells. Outlier threshold is below 10% for recoveries of more than 60 cells. As the number of cells increases, the threshold decreases further, along with the number of outlier variant calls, up to 600 cells where the distribution is very close to sequencer background-noise level.

In summary, these observations show the power of our approach, whereby from cell recoveries as low as 60 cells, setting a variant calling threshold of 10% we get rid of the noise and obtain highly specific and sensitive results. Sensitivity is linked to the fact that we analyze a homogeneous (for marker phenotype and ploidy) population of tumor cells and it is unlikely that a true variant is diluted below the 10% threshold only by effect of sub-clonality within that population and/or wild-type copy gains.

### Dissecting homogenous cell subpopulations

**Figure 5:**
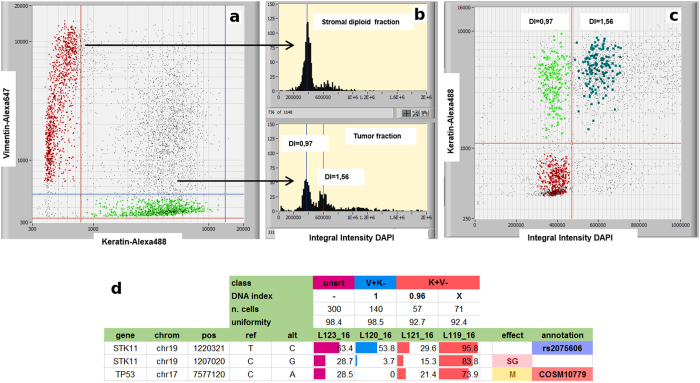
DNA content categories from cells suspension derived from FFPE lung tumor based on multi-parametric analysis. (**a,b**) DNA content histogram show a DNA diploid peak after gating of the V + K cell fraction. K + V- population shows a DNA histogram containing two DNA fractions: a DNA diploid fraction with a DNA index of 0.97 and an hyperdiploid fraction with a DNA index of 1.56. (**c**) DNA-content distribution of cell recoveries. The dots with the same color represent an homogenous cell type, green (K + V- DI = 0.97), dark-green (K + V- DI = 1.56) and red (V + K- DNA diploid). (**d**) Sample S09 frequency profiles show different mutational profiles between stromal, DNA diploid and DNA aneuploid tumor populations. Across the loci displayed (one SNP and two somatic mutations), the DNA aneuploid tumor has a much higher frequency value than the diploid tumor population. SG = stop_gain, M = missense. The variants of the diploid K + V- population (DI = 0,96) confirm its tumor origin.

Qualitative observations of the simultaneous staining of tumor and stromal-associated antigens provided a starting point for quantitative analysis of DNA content^17^, opening up the possibility for the molecular genetic study of intra-tumor heterogeneity^18^ based on DNA content (Fig. 5a–c).

For this purpose, we used DNA index (DI) defined as the ratio of DNA content of a cell population with reference to the DNA content of normal diploid cells. As mentioned above, the DI was measured indirectly through the integral-intensity of DAPI fluorescence, since this is in stoichiometric relationship to the cellular DNA content. Accordingly, to determine the DI for various cell populations we measured the x-axis position of the peaks in the related integral-intensity DAPI histogram.

By definition, a cell population with normal diploid DNA content has a DI of 1.0. Deviations in cellular DNA content values other than 1.0 indicate cell populations containing more (hyperdiploid) or less (hypodiploid) DNA. A cell population with DI equal to 1.0 should properly be referred to as “indistinguishable from DNA diploid” since the DI does not measure the number of chromosomes but only the total DNA content of the cell. However, for simplicity of notation, we refer to tumor cell populations with DI = 1.0 as “DNA diploid” (Table 2 and Supplementary Fig. S4).

In order to assess the reproducibility in determining DI from very small cell numbers, we evaluated the relative standard deviation (RSD) of DI across sample replicates, when available. The small RSD (mean = 3%) observed for both first (n = 38) and second (n = 24) K + V- peaks confirm that DI measure is reliable (Supplementary Table S2).

Sequencing revealed that homogeneous cell recoveries from different tumor subpopulations, sorted by marker phenotype and DNA content, can have genetically distinct profiles, in terms of both somatic mutations and chromosome aberrations.

**Figure 6:**
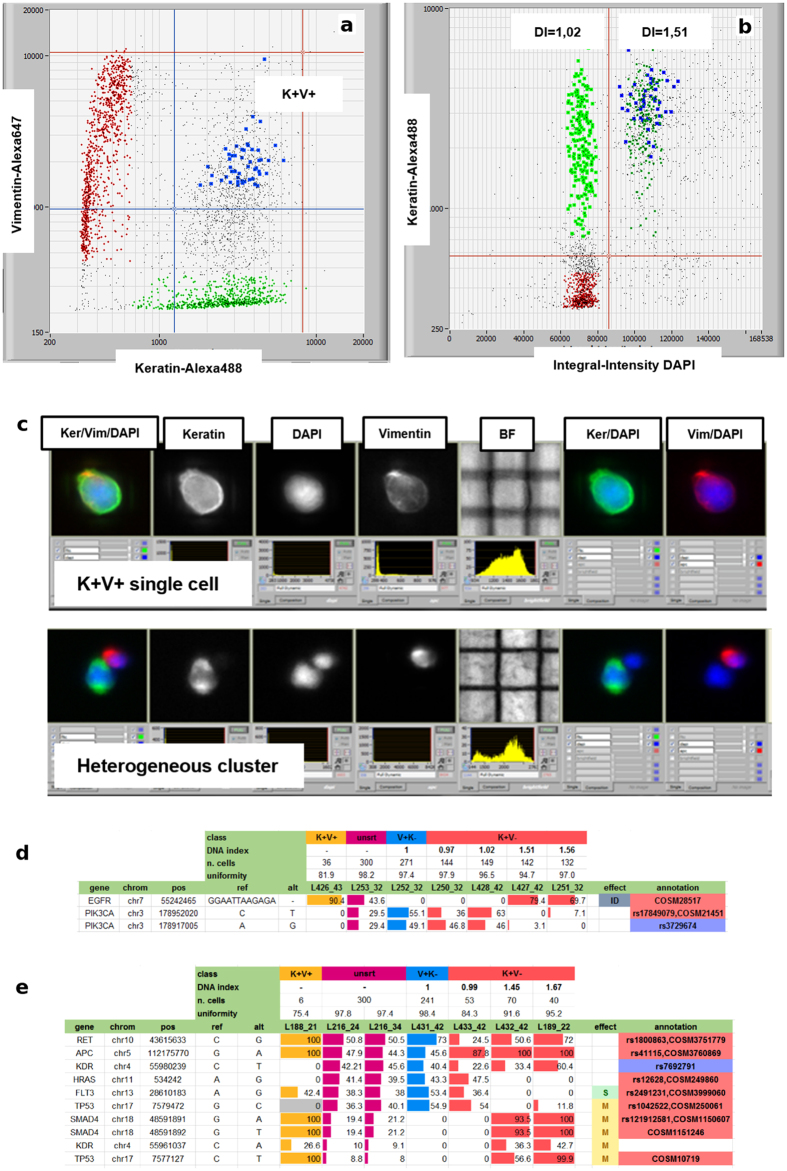
Isolation and genetic analysis of double-positive cells. (**a**) Identification of cells recovered from K + V + population in lung carcinoma sample S11 (blue dots). (**b**) DNA content histogram shows that K + V + cells overlay the DNA aneuploid of the K + V- fraction in the integral intensity DAPI versus keratin dot plot. (**c**) Co-localization of antigens within the same cell is clearly distinguished from heterogeneous clusters. (**d**) Sample S11 variant frequencies highlight a common profile between stromal and diploid K + V- fraction, and between tumor DNA aneuploid and double-positive replicates. ID = in-frame deletion. **e)** Sample S10: *TP53, KDR* and *SMAD4* somatic mutations confirms K + V + recovery comprises tumor cells. S = splice-site, M = missense.

For sample S09 (Fig. 5d), we have two distinct tumor subpopulations, characterized by a different DNA content. Again, due to the concurrent presence of cells coming from normal stroma, a minor DNA diploid and a major DNA hyper-diploid tumor subpopulation, the variant frequencies of the unsorted sample do not allow separation of the genetic profiles for the individual subpopulations. The variants of the DNA diploid K + V- population confirm their tumor origin^19^,^20^ since they are absent from normal stromal cells. As another example, lung cancer sample S11 (Fig. 6d), illustrates how different K + V- cell DNA content corresponds to different genetic profiles, which cannot be resolved from the unsorted sample. In this case, the DNA aneuploid samples clearly show a LOH in the *PIK3CA* locus, with the loss of the variant allele, and -in heterozygosis- a somatic deletion in the *EGFR* locus. Two K + V- diploid replicates have a variant frequency profile consistent with the stromal sample, and lack the somatic *EGFR* deletion detected in K + V- tumor DNA aneuploid cells. Since keratin is not *per se* a specific marker for tumor cells this may imply that those K + V- diploid cells are indeed normal cells, although the alternative interpretation that they are tumor cells but without aberrations covered by the AmpliSeq Cancer HotSpot v2 panel, cannot be ruled out either.

A minor subpopulation of K + V + cells was also observed. Notably, image-based analysis allows us to distinguish precisely keratin/vimentin co-expression by neoplastic epithelial cells from heterogeneous clusters comprised of both V + K- and K + V- cells and resulting for example from incomplete disaggregation (Fig. 6a–c).

Among the K + V + subpopulation, we detected putative somatic variants, different from majority of tumor cells and undetectable in unsorted samples. Since the number of double-positive cells (range = 6–36) sorted from the analyzed sample was below the limit of 60 cells, which we set for specificity reasons in variant calling, we only considered confirmation of tumor origin those somatic variants detected also in the K + V- tumor cells (Fig. 6d,e).

## Discussion

In oncology, precision medicine relies largely on the availability of information about the molecular underpinnings of the tumor biology. NGS holds the potential to make available this information with the accuracy required for clinical implementation. However, to date regulatory clearance for NGS is limited to assays for germline genetic variants. This is due to the inherent trade-off between sensitivity and specificity when the input DNA is a mixture of normal and tumor, and further complicated by the fact that the tumor DNA may derive from subpopulations with different genetic characteristics. This can dilute the signal from the variant alleles or the copy number changes to values too close to the background noise or even below the detection limit.

The method we have developed hinges on pre-analytic digital cell separation from minute FFPE samples. It achieves 100% purity, substantially reverting the DNA composition to a germline-like situation where NGS power works most reliably. Importantly, data analysis and interpretation is also drastically simplified and different classes of genetic alterations can be readily resolved.

Since DEPArray™ system works with a flow-less approach (DEP cages immobilize suspended cells in stable-levitation until selection), samples with down to few thousands or even hundreds of cells can be managed, this in contrast to conventional cell sorters. With the *a-posteriori* gating strategy enabled by DEPArray™, thresholds are set adaptively after scanning the entire sample amenable for sorting. This ensures robustness against inevitable sample-to-sample variations in staining intensity and cellular composition. Purifying subpopulations from polyploid tumors prior to next-generation sequencing analysis addresses the need to characterize tumor heterogeneity at the molecular level.

Optimization of PCR protocol for target enrichment allowed us to push the limit of minimum DNA quantity while preserving results accuracy, as only genetically homogenous subpopulations are sequenced as opposed to a bulk of admixed normal and tumor DNA. Studying the relationship between number of cells, sequencing uniformity and false-positive variant frequencies, we were able to determine operational conditions ensuring high sensitivity and specificity even with FFPE samples, which are notoriously challenging in terms of DNA quality.

Our method renders accessible to accurate molecular genetic analysis specimens with low tumor cellularity, which have been shown to have a poor prognosis^21^,^22^,^23^. Moreover, it may contribute to the study of the phenotypic-genotypic relationships of tumor cell populations. The identification of clinically distinct DNA content sub-groups with different genetic characteristics suggests that a reliable DNA content analysis platform is required and achievable to investigate the complex biological mechanisms that drive cancer.

In addition, the capability of sorting pure stromal cells provides a convenient internal control^24^. Analyzing only the tumor but not matched normal tissue can yield many false-positive alterations that are not specific to the patient’s tumor, including in actionable genes^25^. When matched normal tissue is unavailable, as may be the case for archival samples, our method can provide a valuable surrogate.

## Methods

### FFPE tissue section dissociation

One 50 μm thick section was cut from a formalin-fixed, paraffin-embedded tissue block and collected in a nylon biopsy bag inside a 50 ml conical tube.

The section was dewaxed by three sequential 10 min incubations in xylene and then rehydrated by sequential 5 min incubations in each of the following solutions: 100% ethanol (three times), 70% ethanol (three times), 50% ethanol (two times). The hydration process was completed with 5 min incubations in deionized water.

For Heat-induced antigen retrieval (HIAR), the section was pre-incubated in 10 mM sodium citrate buffer (pH 6.4) for 5 min at room temperature and heat-treated in the same pre-warmed buffer for 1 h at 80 °C. After cooling down at room temperature, the section was washed by three sequential 5 min incubations with RPMI medium (Life Technologies). In order to obtain a cell suspension, the section was incubated in 10 ml of 0.1% collagenase I-A (Sigma-Aldrich) and 0.1% dispase (Life Technologies) mixture diluted in standard RPMI, at 37 °C for 30–45 min in a water bath. Dissociation was monitored by swirling the sample on a vortex every 15 min until the tissue section disintegrate. The dissociation process was stopped by putting the sample tube on ice when the buffer turned cloudy, indicating cell release.

Working on ice, the solution containing cell suspension was resuspended by pipetting and transferred through a 100 μm mesh nylon filter into a 15 ml conical tube. The sample tube containing nylon bag was washed with other 4 ml of ice-cold PBATw (PBS, 0,05% Tween, 1% BSA) and transferred through the same filter.

Cell suspension was centrifuged 5 min at 1,000 g, using a pre-cooled centrifuge (4 °C), to spin down the cells.

Next, the pellet was washed two times with 5 ml of ice-cold PBATw by centrifugation at 1,000 g for 5 min, using a pre-cooled centrifuge (4 °C). The supernatant was discarded and pellet was resuspended in 1 ml of ice-cold PBATw.

### FFPE tissue cores dissociation

Samples were carefully selected based on a routine Hematoxylin and Eosin (H&E) staining from the viable tissue (prior to fixation).

Three to six 0.6 mm tissue cores were taken with a manual tissue arrayer (Estigen OÜ) and stored in an 1.5 mL reaction vessel until further use.

Excess paraffin was removed with a scalpel, tissue cores were cut into small pieces and collected into a gentleMacs Tube C (Miltenyi Biotec GmbH).

Sample was washed three times with 5 ml xylene for 10 min to remove remaining paraffin and then rehydrated in sequential ethanol washes (100%, 5 min, three times; 70% ethanol, 5 min, three times, 50% ethanol, 5 min, two times). The hydration process was completed with 5 min incubations in deionized water.

For Heat-induced antigen retrieval (HIAR), sample was pre-incubated in 10 mM sodium citrate buffer (pH 6.4) for 5 min at room temperature and heat-treated in the same pre-warmed buffer for 1 h at 80 °C. After cooling down at room temperature, sample was washed by three sequential 5 min incubations with plain RPMI medium (Life Technologies).

Dissociation was performed at 37 °C in a water bath using a mixture of 0.1% collagenase I-A (Sigma-Aldrich) and 0.1% dispase (Life Technologies) diluted in standard RPMI. Every 15 min sample was processed using gentleMacs Dissociator (Miltenyi Biotec GmbH) for the mechanical dissociation step (program: m_spleen_03.02 C-tube). Disaggregation was monitored and stopped after 30–45 min putting the sample tube on ice.

Working on ice, the solution containing cell suspension was resuspended by pipetting and transferred through a 100 μm mesh nylon filter into a 15 ml conical tube to remove any remaining larger particles from the single-cell suspension. The sample tube was washed with other 4 ml of ice-cold PBATw (PBS, 0,05% Tween, 1% BSA) and transferred through the same filter.

Cell suspension was centrifuged 5 min at 1,000 g, using a pre-cooled centrifuge (4 °C), to spin down the cells.

Next, the pellet was washed two times with 5 ml of ice-cold PBATw by centrifugation at 1,000 g for 5 min, using a pre-cooled centrifuge (4 °C). The supernatant was discarded and pellet was resuspended in 1 ml of ice-cold PBATw.

### Cell suspension staining

An aliquot of 5 × 10^5^ cells was incubated with 100 μl of primary monoclonal antibody mixture containing anti-keratin MNF116, IgG1 (DAKO) final concentration 3,2 μg/ml, anti-keratin AE1/AE3, IgG1 (Millipore–Chemicon), final concentration 10 μg/ml and anti-vimentin 3B4, IgG2a (DAKO, Glostrup, Denmark), final concentration 3,1 μg/ml for 30 min at 4 °C, in PBATw.

Cells were washed twice with ice-cold PBATw and centrifuged at 1,000 g for 5 min at 4 °C. Then cells were incubated with 100 μl premixed secondary reagents Alexa Fluor® 488 Goat Anti-Mouse IgG1 (Life Technologies) final concentration 2,5 μg/ml for keratin detection, Alexa Fluor® 647 Goat Anti-Mouse IgG2a (Life Technologies), final concentration 2,5 μg/ml for vimentin detection, in PBATw.

After 60 min at 4 °C, cells were washed twice with ice-cold PBATw and incubated, for 30 min at 37 °C, with 0,5 ml DNA staining solution containing 10 μM DAPI (Sigma-Aldrich) in PBATw. After incubation time sample was washed twice with PBATw by a 5 min centrifugation at 1,000 g and pellet was resuspended in the same buffer.

### Ion Torrent PGM library preparation and sequencing

The NGS was conducted by using the Ion AmpliSeq™ Cancer Hotspot Panel v2 (Life Technologies).

Ion Torrent adapter-ligated libraries were constructed with the Ion Ampliseq library kit 2.0 (Life Technologies) according to the manufacturer’s protocol with modifications described as follows. In order to avoid sample loss, targeted PCR reaction Mix was aliquoted directly above the 3 μl of cell lysate. The cycle number was adjusted depending on the number of cells in the input cell lysate. In particular, samples containing a number of cells from 300 to 61, from 60 to 31 and less than 30 were subjected to 23, 25, 28 amplification cycles, respectively.

The resulting amplicons were treated with 2 ul of FuPa reagent to partially digest PCR primers and repair fragment ends. Ion Xpress™ Barcode Adapters were then ligated to the amplicons. After purification with Agencourt® AMPure® XP beads (Beckman Coulter), the ligated DNA underwent 5 cycles of amplification. The resulting library was purified by using the Agencourt® AMPure® XP beads. Library elution volume was adjusted according to the input number of cells; libraries prepared starting from an input number of cells ranging from 300 down to 61, 60 down to 31 and less than 30, were eluted in 40 μl, 30 μl, 20 μl respectively.

The libraries were then quantified by the Qubit ® 2.0 Fluorometer with the Qubit ® dsDNA HS Assay kit (Life Technologies). Agilent® Bioanalyzer® 2100 system with the Agilent® High Sensitivity DNA Kit (Agilent Technologies) was used to analyze the fragment size distribution of each library.

Six to twelve libraries were pooled per each Ion 316™ Chip (Life Technologies) or Ion 318™ Chip (Life Technologies), respectively.

The library templates were prepared according to the “Ion PGM ™ Template OT2 200 kit” user guide.

Ion 316™ Chip or Ion 318™ Chip were loaded following “Simplified Ion PGM™ Chip loading with the Ion PGM™ weighted chip bucket” protocol instructions (MAN0007517).

All samples were processed by Ion Personal Genome Machine (PGM) (Life Technologies) using the Ion PGM™ Hi-Q™ Sequencing Kit (Life Technologies) and setting the 500 flow run format.

### Whole-genome low-pass sequencing for CNV analysis

Recovered cells were lysed in the recovery tube using SB LysePrep™ Kit (Silicon Biosystems) and 46 μl of LowTE buffer were added to the tube. The sample was then transferred into a microTUBE-50 AFA Fiber Screw-Cap for fragmentation by Covaris M220 instrument for 3 min and 52 sec (pick power:50, duty factor:20, cycles/burst:200) to obtain a 150–200 bp fragment size. Libraries were prepared using Accel-NGS® 2S PCR-Free DNA Library kit (Swift Biosciences) according to the manufacturer’s instructions.

20 μl of library were amplified as following: 6 μM of amplicon PCR forward primer (5′-AATGATACGGCGACCACCGAGATC-3′), 6 μM of amplicon PCR reverse primer (5′-CAAGCAGAAGACGGCATACGA-3′) and 2× KAPA HiFi HotStart Ready Mix (Kapa Biosystems). The PCR cycling conditions were 98 °C initial denaturation for 45 sec, followed by 16 cycles and 15 cycles for ~100 cells and ~300 cells, respectively at 98 °C for 15 s, 60 °C for 30 s, and 72 °C for 1 min, and a final extension at 72 °C for 1 min. The products were cleaned up with 0.75X Agencourt AMPure XP beads (Beckman Coulter Genomics) according to the manufacturer’s protocol and eluted in 20 μl Low TE (Swift Biosciences).

Libraries were normalized and pooled to 4 nM based on qPCR quantification. Pooled samples were denatured and diluted to a final concentration of 12 pM. All samples were multiplexed and sequenced in a single lane on the MiSeq using 2 × 100 bp paired-end sequencing using the MiSeq Reagent Kit V3.

### Data analysis

The sequenced data were processed with the Torrent Suite™ v4.2, providing for signal processing, base calling, alignment to Homo sapiens hg19 reference sequence and variant calling. The Torrent Variant Caller was set to somatic low-stringency pipeline, with the following parameters: snp_min_allele_freq = 0.5%, indel_min_allele_freq = 1%, gen_min_alt_allele_freq = 1%, gen_min_indel_alt_allele_freq = 5%. The assessment of true/false positives is then carried out subsequently by the comparative analysis of variant frequency in tumor vs stromal cells. The resulting XLS files were used for comparing variants among different samples, excluding “No call” or “Absent” calls. Mutational events, accounting for frequency variation among cell subpopulations, were predicted using a python script developed for the purpose.

Variant annotation was made by Ensembl Variant Effect Predictor (VEP)^26^,^27^ release 80 using GRCh37.p13 human genome assembly.

Variant report tables summarize a subset of variants. The selection criteria is based on three basic rules, that have to be satisfied independently by both tumor and stromal replicates: a) the mean frequency of a variant in tumor/stromal replicates must be greater than or equal to 10%, b) a variant has to be confirmed by at least 20% of replicates and c) only recoveries with at least 60 cells were considered. Visual inspection by IGV^28^,^29^ was used to clarify uncertain results and exclude obvious false positives, e.g. due to homopolymers or misalignments.

Recoveries with less than 60 cells were used only in special cases to explain some notable events.

For low-pass WGS, we obtained a total of 23 million paired-end reads, 14 million for S01 (2 libraries) and 9 million for S09 (3 libraries). The BWA^30^,^31^ algorithm was used to align the 98.7% reads to the hg19 human reference genome. PCR duplicates and secondary alignments were filtered out using Picard MarkDuplicates^32^ and samtools^33^, obtaining a mean coverage of 0.22× and 0.09× for S01 and S09 sample respectively. Coverage analyses were performed using BEDTools^34^ and custom perl scripts.

Control-FREEC^35^,^36^ algorithm was used to obtain copy-number calls, using the mode without control sample independently for all 5 libraries. Read counts were corrected by GC content and mappability (uniqMatch option) and bins length were determined by software using coefficientOfVariation = 0.06.

Ploidy was set using the formula ploidy = DI*2, where DI means DNA index, calculated as described above. Plots for CNV profiles were obtained using a custom python script.

## Additional Information

Accession codes: NCBI Short Read Archive PRJNA309054 (targeted sequencing). PRJNA309109 (low-pass WGS). 

**How to cite this article**: Bolognesi, C. *et al*. Digital Sorting of Pure Cell Populations Enables Unambiguous Genetic Analysis of Heterogeneous Formalin-Fixed Paraffin-Embedded Tumors by Next Generation Sequencing. *Sci. Rep.* **6**, 20944; doi: 10.1038/srep20944 (2016).

## Supplementary Material

Supplementary Information

## Figures and Tables

**Table 1:** Sample list.

Patient	Sample ID	Sample Source	Sample Type	Tumor Type	Tumor Cellularity	Storage time
1	S01	IEO, Milan	FFPE tissue section 50 μm	Ovarian adenocarcinoma	60%	2 years
2	S02	IEO, Milan	FFPE tissue section 50 μm	Pancreatic-biliary adenocarcinoma	45%	1 year
3	S03	IEO, Milan	FFPE tissue section 50 μm	Pancreatic-biliary adenocarcinoma	30%	1 year
4	S04	LUMC, Leiden	FFPE tissue punches	Lung	5%	3 years
5	S05	LUMC, Leiden	FFPE tissue punches	Lung	30%	1 year
6	S06	LUMC, Leiden	FFPE tissue punches	Lung	30%	1 year
7	S07	LUMC, Leiden	FFPE tissue punches	Lung	40%	1 year
8	S08	LUMC, Leiden	FFPE tissue punches	Lung	30%	1 year
9	S09	LUMC, Leiden	FFPE tissue punches	Lung	40%	1 year
10	S10	LUMC, Leiden	FFPE tissue punches	Lung	5%	1 year
11	S11	LUMC, Leiden	FFPE tissue punches	Lung	30%	1 year
12	S12	LUMC, Leiden	FFPE tissue punches	Lung	30%	1 year
13	S13	LUMC, Leiden	FFPE tissue punches	Rectosigmoid adenocarcinoma	>30%	13 years
14	S14	LUMC, Leiden	FFPE tissue punches	Rectosigmoid adenocarcinoma	>30%	19 years
15	S15	LUMC, Leiden	FFPE tissue punches	Rectosigmoid adenocarcinoma	>30%	21 years
16	S16	LUMC, Leiden	FFPE tissue punches	Rectal adenocarcinoma	>30%	20 years
17	S17	LUMC, Leiden	FFPE tissue punches	Rectal adenocarcinoma	>30%	19 years
18	S18	LUMC, Leiden	FFPE tissue punches	Rectal adenocarcinoma	>30%	20 years
19	S19	LUMC, Leiden	FFPE tissue punches	Rectal adenocarcinoma	>30%	13 years
20	S20	LUMC, Leiden	FFPE tissue punches	Rectal adenocarcinoma	>30%	16 years
21	S21	LUMC, Leiden	FFPE tissue punches	Rectal adenocarcinoma	>30%	20 years
22	S22	LUMC, Leiden	FFPE tissue punches	Rectal adenocarcinoma	>30%	21 years
23	S23	LUMC, Leiden	FFPE tissue punches	Sigmoid adenocarcinoma	>30%	21 years

**Table 2:** DNA Index of the K + cell population.

Sample	Keratin Positive Population		
	DNA Index Peak 1	DNA Index Peak 2	DNA Index Peak 3
Ovarian adenocarcinoma			
S01	1,62	2,70	
Pancreatic-biliary adenocarcinoma			
S02	1,02		
S03	0,97		
Lung cancer			
S04	*		
S05	*		
S06	1,00	1,86	
S07	1,60	2,44	
S08	1,06	1,73	
S09	0,94	1,42	
S10	0,99	1,48	2,40
S11	0,97	1,54	
S12	1,28	2,38	
Rectosigmoid adenocarcinoma			
S13	1,60		
S14	1,24	1,95	
S15	1,44		
Rectal adenocarcinoma			
S16	1,61		
S17	0,98	1,60	
S18	1,48	2,13	
S19	1,68	2,34	
S20	1,36	1,86	2,40
S21	1,00		
S22	1,40		
Sigmoid adenocarcinoma			
S23	1,98		

*DNA Index undetermined. V + fraction not detected. DNA Index value reported is the mean values of replicates.
