# Supplementary material for: Digital Sorting of Pure Cell Populations Enables Unambiguous Genetic Analysis of Heterogeneous Formalin-Fixed Paraffin-Embedded Tumors by Next Generation Sequencing
Source: Sci Rep. 2016 Feb 11;6:20944. doi: 10.1038/srep20944 (PMC4750064; doi:10.1038/srep20944)
Supplement: Supplementary Information [file srep20944-s1.pdf]

Supplementary information to:

Digital Sorting of Pure Cell Populations Enables Unambiguous Genetic Analysis of Heterogeneous Formalin-Fixed Paraffin Embedded Tumors by Next Generation Sequencing

Authors

Chiara Bolognesi[1†] , Claudio Forcato[1†] , Genny Buson[1], Francesca Fontana[1], Chiara Mangano[1], Anna Doffini[1], Valeria Sero[1], Rossana Lanzellotto[1], Giulio Signorini[1], Alex Calanca[1], Maximilian Sergio[1], Rita Romano[1], Stefano Gianni[1], Gianni Medoro[1], Giuseppe Giorgini[1], Hans Morreau[2], Massimo Barberis[3], Willem E. Corver[2], Nicolo Manaresi[1*].

[1]Silicon Biosystems S.p.A, Bologna, Italy;

[2]Department of Pathology, Leiden University Medical Center, Leiden, Netherlands

[3]European Institute of Oncology, Milan, Italy

[†] These authors contributed equally and share first authorship

***Corresponding Author**

Nicolo Manaresi

Silicon Biosystems S.p.A

Via Dei Lapidari, 12

40129 Bologna

Italy

nmanaresi@siliconbiosystems.com

Supplementary Table S1: Sequencing data statistics for sorted cells

Sample ID	Library ID	Recovery Type	n. of Cells	Mapped Reads	On Target	Uniformity	Mean Depth
**S01**	**L12_04**	K+V-	300	556,972	98.4	96.9	2,452
	**L13_04**	K+V-	600	605,716	97.1	98.1	2,617
	**L15_05**	K+V-	300	604,920	96.3	97.0	2,621
	**L16_05**	V+K-	85	558,583	96.6	96.8	2,407
	**L17_05**	V+K-	300	510,532	97.2	97.9	2,215
	**L19_05**	K+V-	85	646,633	96.7	95.9	2,819
	**L20_06**	K+V-	85	669,249	97.3	96.5	2,949
	**L21_06**	K+V-	300	711,014	96.7	97.8	3,143
	**L86_11**	V+K-	25	617,073	98.5	87.2	2,697
	**L87_12**	V+K-	6	463,050	93.4	43.8	1,643
	**L88_12**	V+K-	7	317,212	94.1	60.9	1,291
	**L92_11**	V+K-	23	539,500	98.4	87.7	2,403
	**L93_11**	K+V+	8	648,640	98.0	87.0	2,871
	**L94_11**	K-V-	12	627,647	96.9	82.1	2,741
	**L95_11**	V+K-	25	550,363	98.1	87.5	2,448
	**L96_12**	V+K-	9	446,195	97.9	81.6	1,944
	**L97_12**	K-V-	11	421,468	92.6	71.6	1,665
	**L98_12**	V+K-	8	447,036	94.9	64.5	1,845
**S02**	**L75_10**	V+K-	300	536,046	97.8	98.5	2,428
	**L76_10**	K+V-	121	584,241	98.0	97.5	2,629
**S03**	**L77_10**	K+V-	121	570,224	97.5	97.9	2,520
	**L78_10**	K-V-	91	513,204	96.9	98.1	2,272
	**L79_10**	V+K-	300	630,543	97.4	98.5	2,804
**S04**	**L218_25**	K+V-	300	367,145	98.7	98.3	1,665
	**L219_25**	K+V-	85	354,773	97.7	98.3	1,600
**S05**	**L220_25**	K+V-	300	346,966	98.2	98.7	1,601
	**L221_25**	K+V-	85	402,236	96.6	99.1	1,823
**S06**	**L100_13**	V+K-	300	542,505	95.6	99.0	2,404
	**L101_13**	K+V-	98	619,966	97.8	97.0	2,773
	**L102_13**	V+K-	8	676,990	93.2	80.7	2,712
	**L103_13**	K-V-	5	684,948	94.2	71.6	2,850
**S07**	**L105_14**	K+V-	100	649,669	96.9	95.2	2,873
	**L106_14**	V+K-	275	644,751	97.4	99.0	2,876
	**L107_14**	V+K-	24	672,177	96.5	81.7	2,789
	**L108_14**	K+V+	18	685,298	95.6	74.4	2,751
	**L183_21**	V+K-	7	362,521	93.5	60.2	1,522
	**L184_21**	K+V+	10	435,464	90.8	50.5	1,682
	**L185_21**	K+V-	60	469,387	96.7	92.7	2,057
	**L186_21**	K+V-	280	566,798	97.0	92.4	2,473
	**L187_21**	V+K-	184	470,899	97.1	96.9	2,081
	**L200_23**	V+K-	266	578,947	96.8	96.6	2,566
	**L201_24**	K+V-	273	536,202	96.6	92.5	2,356
	**L203_24**	K+V-	285	537,397	96.7	94.4	2,369
	**L209_23**	V+K-	110	515,536	96.2	96.1	2,276
	**L210_24**	K+V-	229	485,917	95.6	95.3	2,115
**S08**	**L111_15**	K+V-	21	509,581	95.8	84.0	2,166
	**L112_15**	K-V-	57	404,163	97.3	89.2	1,751
	**L113_15**	K+V+	17	516,207	96.1	78.6	2,188
	**L114_15**	V+K-	299	398,804	97.3	92.7	1,694
	**L115_15**	V+K-	84	322,235	97.7	96.4	1,431
	**L116_15**	V+K-	34	454,616	95.5	73.6	1,803
	**L423_42**	K+V-	304	268,883	95.6	98.5	1,166
**S09**	**L118_16**	K+V-	193	537,783	97.5	95.0	2,410
	**L119_16**	K+V-	71	552,828	97.5	92.4	2,441
	**L120_16**	V+K-	140	552,212	97.3	98.5	2,470
	**L121_16**	K+V-	57	507,266	97.4	92.7	2,242
**S10**	**L188_21**	K+V+	6	440,359	94.5	75.4	1,817
	**L189_22**	K+V-	40	467,814	97.1	95.2	2,062
	**L431_42**	V+K-	241	250,791	96.8	98.4	1,109
	**L432_42**	K+V-	70	252,339	96.1	91.6	1,096
	**L433_42**	K+V-	53	237,460	97.7	84.3	1,041
**S11**	**L250_32**	K+V-	144	191,380	95.6	97.9	841
	**L251_32**	K+V-	132	232,308	95.4	97.0	1,008
	**L252_32**	V+K-	271	209,420	95.3	97.4	915
	**L426_43**	K+V+	36	569,280	98.8	81.9	2,459
	**L427_42**	K+V-	142	253,089	96.7	94.7	1,122
	**L428_42**	K+V-	149	257,380	96.1	96.5	1,142
**S12**	**L192_22**	K+V-	85	435,944	97.3	96.0	1,982
	**L193_22**	K+V-	44	531,044	98.0	94.3	2,392
	**L194_22**	V+K-	85	477,375	97.7	97.6	2,187
	**L195_22**	V+K-	20	615,017	97.5	92.6	2,774
	**L196_22**	K+V-	28	564,587	97.8	91.8	2,574
**S13**	**L312_37**	K+V-	235	563,637	96.6	96.4	2,441
	**L319_36**	V+K-	160	314,857	94.8	96.4	1,341
**S15**	**L398_42**	V+K-	191	244,028	98.8	96.2	1,101
	**L399_44**	K+V-	292	487,522	98.6	94.9	2,189
**S16**	**L320_36**	V+K-	160	441,694	95.3	97.9	1,889
	**L322_37**	K+V-	143	578,834	95.3	98.4	2,480
	**L328_37**	K+V-	47	645,526	97.1	93.8	2,820
	**L330_36**	K+V-	43	368,168	95.8	90.4	1,595
	**L439_43**	K+V-	59	494,375	94.3	90.2	2,094
	**L530_52**	V+K-	165	430,552	96.5	98.5	1,861
	**L531_52**	K+V-	61	392,091	96.6	92.6	1,683
	**L532_52**	K+V+	17	408,264	94.3	82.6	1,710
**S17**	**L310_36**	K+V-	297	373,809	94.9	96.9	1,567
	**L313_36**	V+K-	233	357,368	95.5	97.0	1,518
	**L332_36**	K+V-	27	327,526	96.2	66.8	1,294
**S18**	**L297_36**	K+V-	15	444,354	92.1	67.2	1,792
	**L299_36**	K+V-	231	363,140	92.8	96.0	1,488
	**L300_36**	V+K-	242	320,453	93.1	90.8	1,302
**S22**	**L436_43**	V+K-	220	474,070	97.6	94.9	2,037

Supplementary Table S2

Sample ID	#Replicates Peak 1	Mean DI Peak 1	SD Peak 1	RSD Peak 1	#Replicates Peak 2	Mean DI Peak 2	SD Peak 2	RSD Peak 2
**Ovarian adenocarcinoma**								
S01	10	1,62	0,05	3%	9	2,70	0,07	2%
**Pancreatic ductal adenocarcinoma**								
S02	2	1,02	0,07	7%				
S03	2	0,97	0,02	2%				
**Lung cancer**								
S07	6	1,60	0,07	4%	2	2,28	0,27	12%
S08	3	1,06	0,03	2%	3	1,73	0,05	3%
S09	2	0,94	0,03	3%	2	1,42	0,02	1%
S10	3	0,99	0,01	1%	3	1,48	0,02	1%
S11	3	0,97	0,04	4%	3	1,54	0,03	2%
**Rectal adenocarcinoma**								
S16	3	1,61	0,09	5%				
S19	2	1,68	0,05	3%	2	2,34	0,06	2%
S22	2	1,40	0,02	1%				
	**# Replicates**	**DI Mean**	**SD Mean**	**RSD Mean**	**# Replicates**	**DI Mean**	**SD Mean**	**RSD Mean**
	38	1,26	0,04	3%	24	1,93	0,07	3%

Supplementary Table S2: Relative standard deviation (RSD) of keratin-positive fraction DNA Index across sample replicates.

Relative standard deviation describes the amount of variability relative to DNA Index value observed in replicates of the same sample and is defined as the ratio of the standard deviation to DNA Index reported as mean values of replicates, expressed in %. DNA content replicates analysis was performed for 11 samples, and DNA Index was calculated for each integral intensity DAPI histogram for both first and second peak if present.

The first peak represents DNA diploid and DNA aneuploid fraction with DI mean = 1,26 (range 0,94-1,68; SD = 0.04) evaluated across 38 replicates. In 7 out of 11 samples a second DNA aneuploid peak was recorded with DI mean=1,93 (range 1,42-2,7; SD = 0,07) estimated across 24 replicates.

The mean RSD = 3%, observed for both first and second peak positions, confirms the low variabilty between DI replicates measurements and supports the reproducibility and reliability of the method.

Supplementary Table S3

Sample ID	Tumor Type	Tumor Cellularity	gene	chrom	position	ref allele	alt allele	unsrt	tumor	HGVS notation	protein effect	annotation
S10	Lung	5%	**SMAD4**	chr18	48.591.891	GG	AT	19,4	93,5	**p.G352I**	missense_variant	rs121912581,COSM1150607,COSM1151246
			**KDR**	chr4	55.961.037	C	A	10	36,3	**p.S968I**	missense_variant	-
			**TP53**	chr17	7.577.127	C	T	8,8	56,6	**p.E271K**	missense_variant	COSM10719
S03	Pancreatic ductal adenocarcinoma	30%	**KRAS**	chr12	25.398.285	C	G	3,1	18,2	**p.G12R**	missense_variant	rs121913530,COSM518
			**TP53**	chr17	7.577.022	G	A	2,8	30,1	**p.R306***	stop_gained	rs121913344,COSM10663
S06	Lung	30%	**BRAF**	chr7	140.453.136	A	T	35,2	55,3	**p.V600E**	missense_variant	rs113488022,COSM476
S08	Lung	30%	**EGFR**	chr7	55.242.465	GGAATTAAGAGAAGC	-	28	53,9	**p.E746_A750del**	inframe_deletion	rs121913421,COSM6223
S11	Lung	30%	**EGFR**	chr7	55.242.465	GGAATTAAGAGAAGC	-	43,3	69,6	**p.E746_A750del**	inframe_deletion	rs121913421,COSM6223
S12	Lung	30%	**KRAS**	chr12	25.398.284	C	T	49,3	54,5	**p.G12D**	missense_variant	rs121913529,COSM521
S16	Rectal adenocarcinoma	>30%	**KRAS**	chr12	25.398.284	C	A	37,3	55,1	**p.G12V**	missense_variant	rs121913529,COSM520
			**PIK3CA**	chr3	178.936.082	G	A	21,5	43,1	**p.E542K**	missense_variant	rs121913273,COSM760
S07	Lung	40%	**STK11**	chr19	1.220.400	G	T	88,8	100	**p.E165***	stop_gained	COSM48902
S09	Lung	40%	**TP53**	chr17	7.577.120	C	A	28,5	82,9	**p.R273L**	missense_variant	rs28934576,COSM10779
			**STK11**	chr19	1.207.020	C	G	28,7	88,2	**p.Y36***	stop_gained	-
S02	Pancreatic ductal adenocarcinoma	45%	**TP53**	chr17	7.577.085	C	T	38,8	35,4	**p.E285K**	missense_variant	rs112431538,COSM10722
			**KRAS**	chr12	25.398.284	C	A	28	34,6	**p.G12V**	missense_variant	rs121913529,COSM520
S01	Ovarian adenocarcinoma	60%	**TP53**	chr17	7.578.461	C	A	60,6	94,5	**p.V157F**	missense_variant	rs121912654,COSM10670

Supplementary Table S3: Summary of clinically relevant somatic mutations detected in sorted tumor cells.

Table shows samples, ordered by increasing tumor cellularity, along with allele frequency of clinically relevant non-synonimous somatic mutations detected in sorted tumor fraction, compared to unsorted mixed cells. Gene/protein effect are described in the "protein effect" column and variants annotated in COSMIC (C) and/or dbSNP (rs) databases are represented in "annotation" column. Clearly, the lower the tumor cellularity the higher the advantage of analyzing sorted cells as we propose in our workflow.

In sample S10, despite the enrichment for tumor cells in the highly intermingled samples (FFPE core punches 0,6 mm diameter), cancer mutations occur at low frequencies in unsorted fraction because of contamination from normal cells or tumor heterogeneity, whereas in sorted tumor cells the real zygosity state is detected.

Resolution of genomic data from heterogeneous samples is evident also in sample S09 with 40% tumor cellularity where, in unsorted fraction, stromal contamination results in diluted frequencies compared to sorted tumor cells.

Supplementary Figure S1

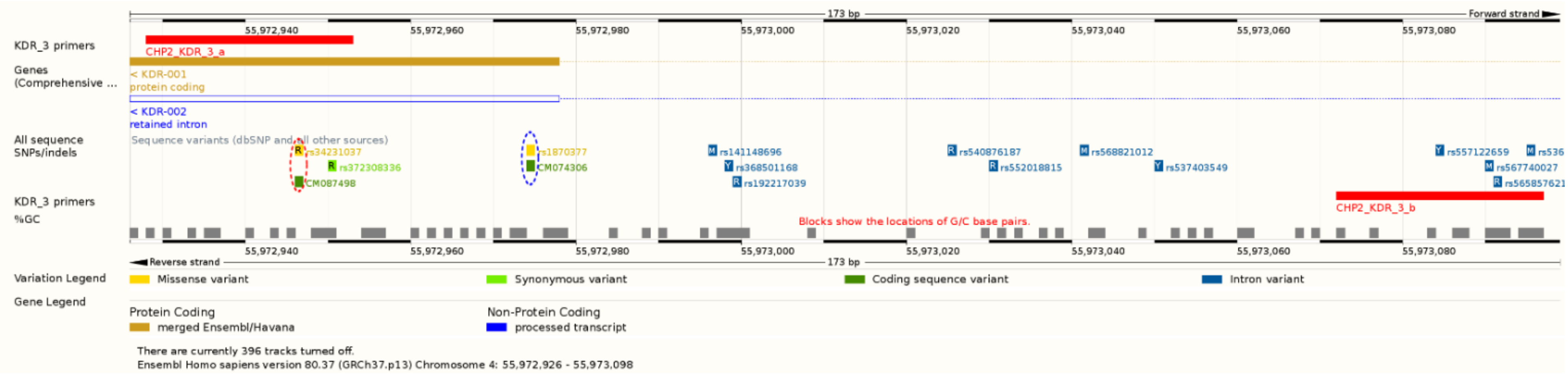

Supplementary Figure S1: SNP position in correspondence of one of the *KDR* targeted sequencing primers

This screenshot, taken from ENSEMBL Genome Browser (http://www.ensembl.org), shows the genome region of the *KDR* mutation (Fig. 3 row 16 – dashed blue circle). In the figure multiple tracks are listed providing information about the presence of known variants in the primers of *KDR* amplicon. In particular, "*KDR_3 primers*" track displays the amplicon primers with red bars and "*All sequence SNPs/indels*" track shows all known variants. The rs34231037 (dashed red circle) is the most relevant variant (between all those falling inside primers) as it has a global minor allele frequency (GMAF) of 0.009, near the threshold (0.01) to be considered a polymorphism.

Supplementary Figure S2

				class		unsrt	V+K-	K+V-					
				DNA index		-	1	DIx					
				n. cells		300	300	121					
				uniformity		99.0	98.5	97.9					
	gene	chrom	pos	ref	alt	L215_24	L79_10	L77_10	GVC	event	effect	annotation	gmaf
1	APC	chr5	112175770	G	A	100	100	96.6		grm HOM	synonymous_variant	rs41115,COSM3760869	G:0.3345
2	FGFR3	chr4	1807894	G	A	100	100	100		grm HOM	synonymous_variant	rs7688609	G:0.0439
3	PDGFRA	chr4	55141055	A	G	100	100	100		grm HOM	synonymous_variant	rs1873778,COSM1430082	A:0.0423
4	FLT3	chr13	28610183	A	G	56.2	49	52		grm HET	splice_region_variant	rs2491231,COSM3999060	A:0.4367
5	EGFR	chr7	55249063	G	A	55.4	69.3	39.2		grm HET	synonymous_variant	rs1050171,COSM1451600	A:0.4327
6	KDR	chr4	55972974	T	A	54.2	56.2	50.7		grm HET	missense_variant	rs1870377,COSM149673	A:0.2119
7	IDH1	chr2	209113192	G	A	49.9	46.4	53.8		grm HET	synonymous_variant	rs11554137,COSM1741220	A:0.0569
8	RET	chr10	43613843	G	T	48.8	56.4	49.1		grm HET	synonymous_variant	rs1800861	G:0.2875
9	KDR	chr4	55980239	C	T	44.9	34	51.1		grm HET	intron_variant	rs7692791	C:0.4559
10	KDR	chr4	55962546	-	G	44.5	41.28	44.5		grm HET	intron_variant	rs3214870	G:0.2410
11	TP53	chr17	7579472	G	C	44	58.6	25.9		grm HET CNVg	missense_variant	rs1042522,COSM250061	G:0.4571
12	KRAS	chr12	25398285	C	G	3.1	0	18.2		som HET	missense_variant	COSM518,COSM1157797	-
13	TP53	chr17	7577022	G	A	2.8	0	30.1		som HET	stop_gained	rs121913344,COSM10663	-
14	CSF1R	chr5	149433596	T	-	0	100	100					
15	CSF1R	chr5	149433598	-	A	0	100	98.4		grm HOM	3_prime_UTR_variant	rs2066934;rs2066933	T:0.2033;G:0.2041
16	CSF1R	chr5	149433596	TG	GA	98.5	0	0					

Supplementary Figure S2: Dilution of somatic variants in unsorted fractions.

Summary of variant frequencies found in S03 patient, affected by pancreatic biliary adenocarcinoma (tumor cellularity 30%). Table shows variants in rows and cell populations in columns (unsrt, unsorted cells; V+K-, diploid stromal cells; K+V-DIx, tumor cells with mixed ploidy). Variants for each cell population are represented by a cell box filled with different colors based on relative frequency: red for tumor, blue for stromal, magenta for unsorted. In the table header, information about number of cells and sequencing uniformity are present. The frequency patterns highlight different kind of variant events summarized in a colored box (column GVC, Genetic Variant Class): somatic heterozygous variant (black), copy number gains (green) and germline variants (ice). In the right-side, the “effect” column describes gene/protein effect, the “annotation” column shows the COSMIC and/or dbSNP IDs and the “gmaf” column indicates the population frequency of the minor allele.

Row 12 (*KRAS*) and 13 (*TP53*) describe two somatic variants clearly detectable in sorted cells, but not easily distinguishable from the background-noise in the unsorted fraction.

Row 14-16 refer to the same variant, as they are close each other and with similar frequencies. A manual inspection of the raw data confirmed the hypothesis, and different calls are due to read mis-alignments caused by homopolymers in this genome region.

Supplementary Figure S3

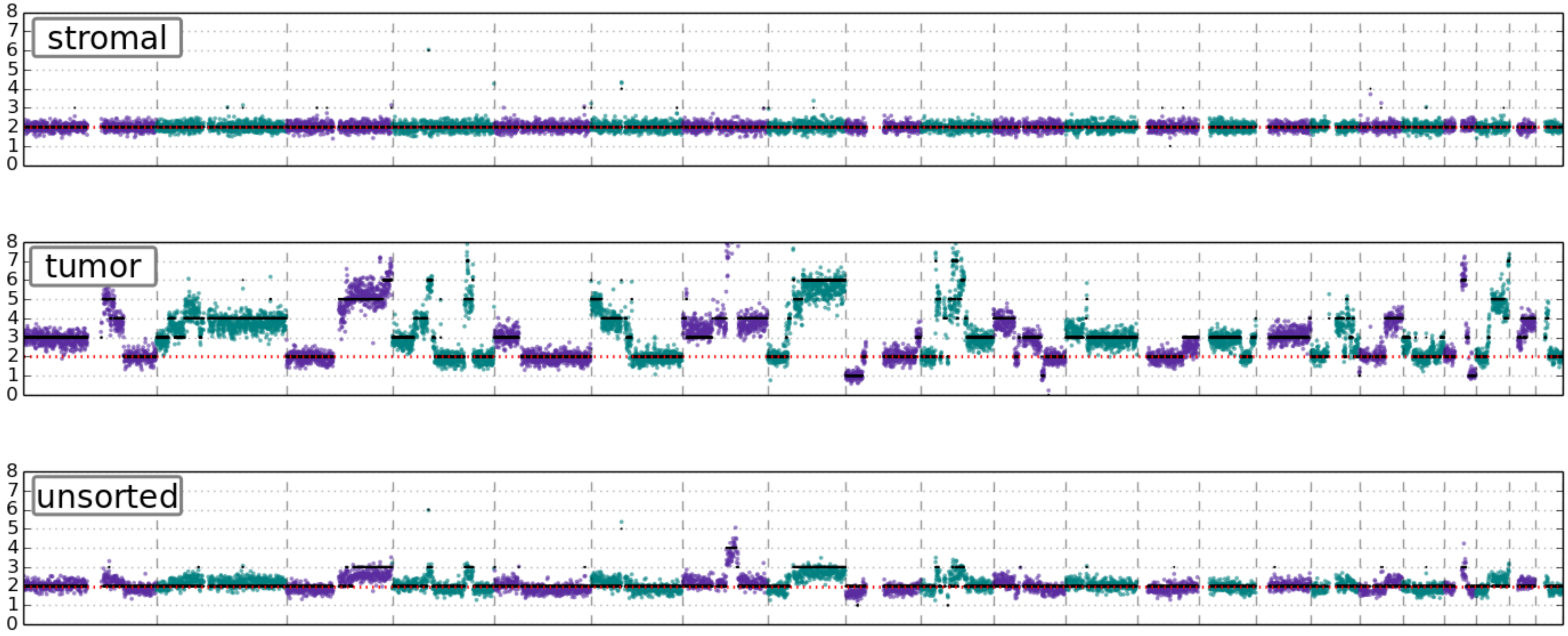

Supplementary Figure S3: Copy-number profiles obtained by low-pass whole genome sequencing of S09 sample.

Whole-genome profiles (chr1-22) of stromal (top), tumor (center) and unsorted (bottom) populations. It is worth to note that tumor population is centered on ploidy=3, as with DEPArray™ sorting is possible to determine DNA content and figure out a near-true ploidy. This is more complicated with unsorted samples.

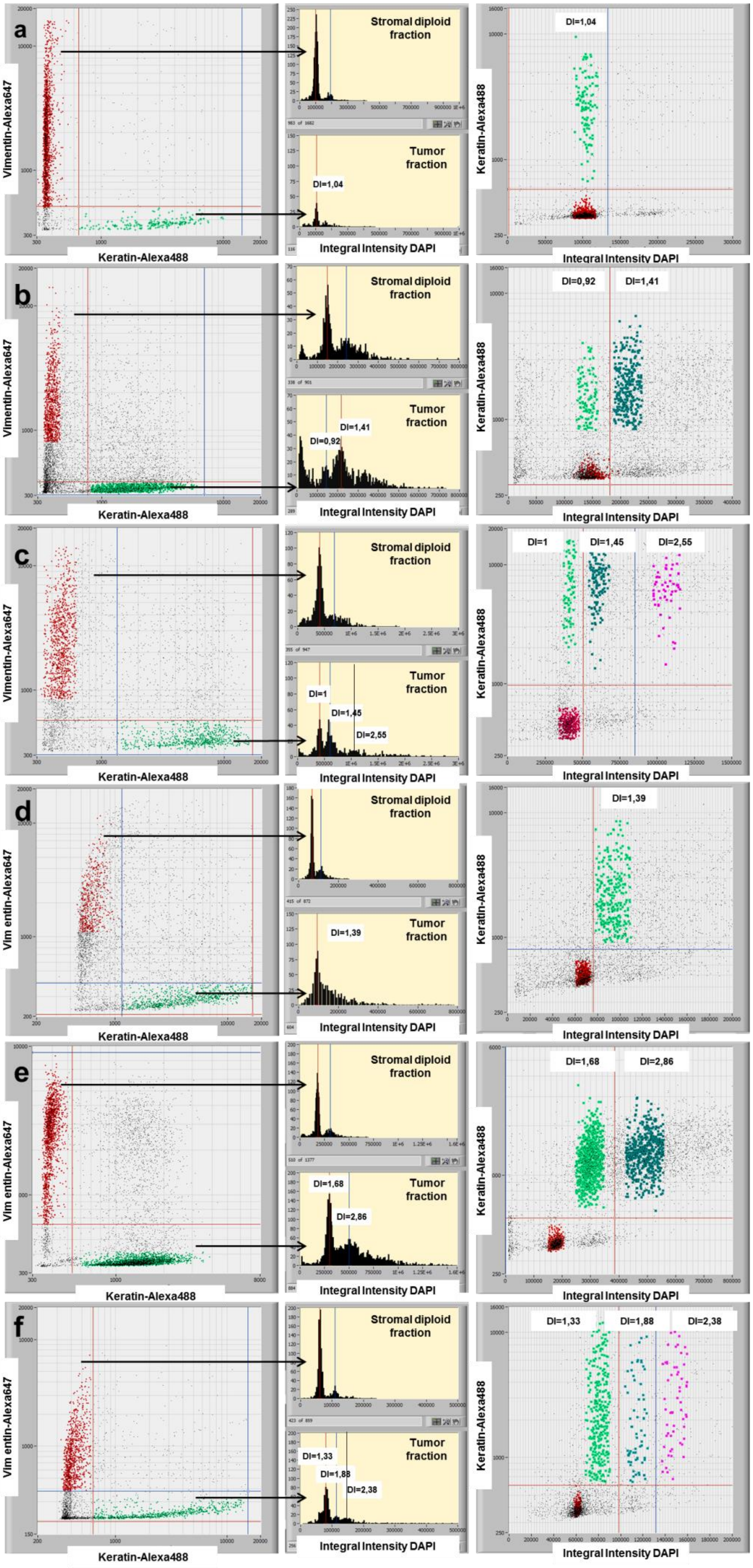

Supplementary Figure S4: DNA Index of the Keratin-positive cell population based on multi-parametric analysis.

For each sample: keratin-Alexa488 / vimentin-Alexa647 scatter plot with highlighting in red of stromal V+K- population and in green of tumor K+V- population (left), integral-intensity-DAPI histograms (mid) for V+K- population (top) and K+V- population (bottom), integral-intensity-DAPI / kertain-Alexa488 scatter plot with highlighting of recovered cells (right).

In 21 of 23 samples (91%) a K+V− cell population as well as a V+K− cell population could be clearly identified. After gating on the V+K− cell fraction a DNA diploid peak is observed allowing tumor fraction DNA measurement.

(a) In 3 of the 21 carcinomas, tumor cell populations shows a DNA peak overlapping with the normal DNA-diploid peak of the V+K− cells population. It should be noted that the absence of an abnormal DNA content does not exclude the existence of an abnormal karyotype, such as a balanced translocation. (**b**) In five lung carcinomas and in one rectal adenocarcinoma the dominant left-most peak of the tumor cells DNA histogram overlaps with the DNA diploid peak of the V+K− cell fraction but an additional hyperdiploid peak is present.**(c)** In one lung cancer sample the tumor cell population shows a DNA diploid peak (DI = 0,99) and two hyperdiploid peaks (DI = 1,48, DI = 2,4). **(d)** In five of the twelve DNA aneuploid samples only one dominant DNA aneuploid population is present**.** (**e**) Six carcinomas show two DNA aneuploid tumor cell populations with a prevailing first peak with DI mean=1.48 and a second peak with DI mean = 2.32. **(f)** In only one rectal adenocarcinoma, 3 different hyperdiploid tumor cell populations are detected (DI = 1,36, DI = 1,86, DI = 2,4).
